# Moral and Physical Massage

**Published:** 1887-04-16

**Authors:** Stretch Dowse


### Moral and Physical Massage.

By Dr. Stretch Dowse.

A Lecture delivered at the Midwives' Institute and Trained Nurses' Club, Buckingham Street.

I will now tell you something about the effects of massage, and I shall then complete the last half-hour of my lecture by practically demonstrating the various processes of massage, and how best to perform them. In speaking to you of the effects of massage, I am going over ground that I have, in part, gone over before in my first lecture,* but I did not complete that part of my subject. I may say that, as a rule without variation, the night process of massage has a calm, soothing, tranquillising and composing effect. It is a very common thing for a patient to say, if it were not for the comforting effect of massage, I feel quite sure from my sense of restlessness and irritability I should have a bad night; but the next morning, upon inquiry, I find the result has been just that which was anticipated. That massage allays a conscious sense of irritability, and brings about a soothing and calming influence, is a matter of everyday experience, and, I may also say, a matter of history. Massage is a tonic; it invigorates, strengthens, animates the dormant powers and energies; it undoubtedly raises the temperature of the body generally. The temperature may fall slightly at first, as I have repeatedly found; but this is not from the effect of massage, it is due to some part of the body being exposed, and heat is thus lost by refraction. The effect of massage is to raise the temperature and improve the circulation, two of the most important points in cases of nervous exhaustion, where the feet and hands are usually icy cold and the pulse so ridiculously small and weak that at the wrist it can scarcely be appreciated.

In a patient, a young man whom I had under my care, the pulse at the wrist was so small, soft, and weak that it could not be noted before massage, but at the end of the *séance* it was felt quite distinctly. After a week's massage its presence could be detected at any time, although still weak. At the end of six weeks the pulse was full, about eighty to the minute, and the extremities were warm and comfortable. In cases of nervous exhaustion the respirations, like the pulse, are shallow and feeble, and sighing and yawning are frequent and common, and absolutely uncontrollable. After massaging the intercostal muscles and nerves, the respirations become deep and regular. The effect of bodily massage in improving nutrition, in cleaning the tongue, in regulating the bowels, and in clearing up the urine, are matters of everyday observation.

We will now consider some of the more practical and physiological results of massage. Dr. Friedrich Busch, whose excellent work on general Orthopœdics, Gymnastics, and Massage, which has been translated by Mr. Noble Smith, writes as follows about absorption by massage:—"As concerns the hastening of the absorption, we know from the experiments carried out for this purpose by Von Mosengeil, Lassar, Generisch, Paschutin, and others, that centripetal rubbings, pressings, kneadings, as well as movements on the joints, hasten the lymph stream, and thus conduct the collected fluids more rapidly into the tissues. This experiment is quite confirmed in man also. Extravasations and exudations disappear more quickly when they are submitted to this mechanical treatment than when left alone. The increased surface of absorption by means of the dispersal of the collected fluid constituents over a larger area, their conduction towards the centre by the centripetal rubbings, the suction-pump action which the muscular movement effects, the increased flow of blood which occurs after every massage, and which exhibits itself by redness of the skin and elevation of the local temperature, are the principal elements of the treatment. The effect of massage in acute muscular pains and spasm of the neck (torticollis), and of the muscles of the loins (lumbago), is such that physicians are now beginning to recognise the fact, and it is found that these pains often disappear more quickly by massage, aided by stimulating liniments, than they do by repose, bandaging, cupping, blisters, injections of morphia, etc.

I think it would be well to consider here the effects of massage upon sprained joints. Sprains of joints are so common and massage is so useful, that I would advise you to give your close attention to what I am going to say in reference to this matter. When a joint is moved beyond its normal range of motion by some external force, the tendons and ligaments are severely strained and stretched, if they are not actually torn, and the articular surfaces of the bones are displaced. When this condition exists, the joint is said to be sprained. It is a common saying that a sprained joint is longer getting well than a broken bone, and this is perfectly true, because sprains, as a rule, are very badly treated. I have alluded to this in my first lecture, but I will treat it more exhaustively now. You must, as I have told you, study the anatomy of joints, and note the position of parts in relation to the joint, and then, if you have to deal with a joint injury, you will see how far the joint is distorted, and you will see, when you have placed each part in its proper position. The readjustment of these parts may be looked upon as the first step in the massage process. There are some people who hold to the opinion that a sprained joint may be lightly bandaged and massaged straight on every day until it is well. My experience leads me to the conclusion that a sprained joint, such as the ankle, or the knee, or the wrist, or elbow, should be thoroughly extended, and when the parts are readjusted it should be very carefully, but very completely and thoroughly, strapped. Then the patient should be allowed to use it; but, remember, the joint should be encased in strapping, so that no yielding can take place. After a week or ten days, the plaister should be removed and massage commenced, and the joint itself manipulated. Every over-stretched muscle becomes by the strain cramped and tense, and it is these painful muscular tensions which yield to the influence of soft rubbings,

* *On the Modern Treatment of Disease by the Process of Massage.* By Thomas Stretch Dowse, M.D., F.R.C.P.E. Messrs. Griffith, Farren, O'Reden, and Walsh, St. Paul's Churchyard.

in the same way that lumbago does. "The mode of manipulation" will be demonstrated in the next lecture. After a few operations the movements of the foot are tolerably free, and possibly painless, and then the patient may be permitted to use it with care, continuing the massage, as before, every day until it has resumed its normal strength. Whilst your attention is directed to the subject of joints, I must not forget to speak to you about the massage and movement required in the breaking down of adhesions which form usually in the parts outside of the joint during the course of the inflammation, especially where the joint has been left alone for weeks. Hot spray and steam douches assist in softening these adhesions, whilst the masseuse manipulates by rubbing, pressing, kneading, and, as Busch says, when the time has come for it, also by passive movements. The masseur proceeds slowly until mobility gradually becomes freer, and more active movements are possible. The ultimate cure of these cases depends upon the experience, the natural dexterity, and even strength of the masseur.

You must remember that adhesions of all parts are most common after acute and active inflammation. These may occur in the membranes of the brain and spinal cord, the sheaths of nerves, the connective tissue of muscle, the membranes of the eye and the ear, the covering of the lungs and the heart, of the liver, the intestines, the ovaries, and the uterus. How far these adhesions can be broken down by movement and by massage it is difficult to say, yet it is astonishing how much can be done by patience, perseverance, and skill. There is always a natural tendency of displaced parts to right themselves and regain their normal position, and massage is unquestionably a powerful auxiliary.

Now, if you please, we commence the business of this lecture. We have come to consider massage proper, that is, to the definition and demonstration of the modes of manipulation and the degrees of energy imparted from the masseur to the patient. I am not going into the question of what is and what is not the difference between vitalised energy and energy imparted by mere mechanical aid. All that I care to know is this, and I am speaking from a large experience, that the two things in their effects are as distinct as the vitalising agency of the sun in comparison with that of the moon. The patient being properly prepared, the masseur or masseuse appears upon the scene, lightly clad, with the arms bare above the elbows, fresh, vigorous, active, bright, and cheerful, showing a determination and a will to do battle with and conquer a hidden but a truly deadly foe. Please to understand me once more, the will of the masseur must be invulnerable; there must be no doubt, no hesitancy, no dread, no fear; the energy imparted must be accompanied with a sustained, innate, conscious effort of will and resolution to overcome every difficulty. If you commence the battle of massage in this way, depend upon it the victory is secure. If, on the other hand, you go about your work tired, indifferent, and doubtful, you only do harm to your patient instead of good, and your downfall is certain. There are four terms which are expressive of the modes of imparting energy by manipulations or massage, and these drawings will show you, and the position of my own hands will show you, how these processes of manipulating can be most effectually carried out. The four terms are—*effleurage, petrissage, tapotement, friction.* Before proceeding to demonstrate these manipulations upon the human body, I will first call your attention to these four pieces or layers of dough. No. 1 was prepared Tuesday, No. 2 was prepared Wednesday, No. 3 was prepared Thursday, and No. 4 was prepared this morning. If you feel these pieces of dough, you will find that they differ in what we call consistency. Now my object is to bring these pieces of dough together and weld them, so to speak, into one homogeneous whole. Now, in effecting this, I shall so manipulate the dough that I shall bring every form of massage to bear upon it to ensure its transformation. Here, you see, is effleurage, here petrissage, here friction, and here tapotement, with the result as you see. I now have a mass of dough of like consistency throughout. I have thus transformed inconsistency and heterogenity into consistency and homogenity by massage. I will now call your attention to this lemon. It is, as you know, a succulent fruit, of uniform consistency. If I submit it, as I now do, to petrissage, by pinching it with my finger and thumb, and also with the muscles of my hand, and tapote it with my clenched fist, I produce great changes in the structure of the lemon. You see before massage its wall or skin covering was tough and unyielding; now you see it yields to effleurage, or mere stroking with the tips of the fingers. I now cut it open, and what do you see? the organisation of the lemon completely broken. Such has been the energy which I have expended upon this fruit, that I have overcome its normal power of resistance, broken up and disintegrated its contents, and by what? By massage. These demonstrations are very simple, and they may appear almost ridiculous, but they are teaching, nevertheless.

(*To be continued.*)

